# Beating the heat: ecology of desert bobcats

**DOI:** 10.1186/s12862-022-01973-3

**Published:** 2022-03-04

**Authors:** John Draper, Torrey Rodgers, Julie K. Young

**Affiliations:** 1 Ecology Center, Utah State University, Logan, UT 84322 USA; 2 Present Address: Department of Wildland Resources, Utah State University, Logan, UT 84322 USA; 3 U.S. Department of Agriculture, National Wildlife Research Center - Predator Research Facility, Millville, UT 84326 USA

**Keywords:** Bobcat, *Lynx rufus*, Meta-barcoding, Home range, Resource selection, Habitat selection

## Abstract

**Background:**

Relative to temperate regions, little is known about bobcats (*Lynx rufus*) in the Sonoran Desert portion of their range, in part due to the difficulty of sampling an elusive carnivore in harsh desert environments. Here, we quantify habitat selection and evaluate diet of bobcats at Kofa National Wildlife Refuge, Arizona, USA, using multiple sampling techniques including GPS telemetry, camera traps, and DNA metabarcoding.

**Results:**

Home ranges during the hot season were smaller than during the cool season. Camera trapping failed to yield a high enough detection rate to identify habitat occupancy trends but third-order resource selection from GPS-collar data showed a preference for higher elevations and rugged terrain at lower elevations. Diet composition consisted of a diverse range of available small prey items, including a higher frequency of avian prey than previously observed in bobcats.

**Conclusions:**

Desert bobcats in our study maintained smaller home ranges and primarily consumed smaller prey than their more northern relatives. This study illustrates the benefit of employing multiple, complementary sampling methods to understand the ecology of elusive species.

**Supplementary Information:**

The online version contains supplementary material available at 10.1186/s12862-022-01973-3.

## Background

Information on ecological traits of carnivores, such as habitat use, home ranges, and diet, can improve our understanding of necessary habitat and prey requirements for conservation and management. These traits may depend on whether the species is a specialist or generalist [1]. While generalists exhibit flexible patterns in these ecological traits, some species, like bobcats (*Lynx rufus*), need habitat with sufficient cover to allow for successful stalking and capture of preferred prey species and home ranges large enough to provide adequate hunting grounds [1–3].

Bobcats occur from northern British Columbia in Canada to central Mexico and have been most heavily studied in the northern portion of their range, where they show a clear preference for forested habitats [4–6]. In the northern and more mesic portions of their range, forested habitats provide suitable hunting grounds for stalking and ambush hunting [7]. In the more xeric southern portions of their range, where forested habitat is rare, bobcats tend to prefer wetlands when available or dry washes that provide cover to aid in prey stalking and ambush [3]. However, limited information exists on habitat use by bobcats in deserts, especially those lacking wetlands.

Bobcats display temporal movement patterns linked to prey vulnerability and temperature, whereas home-range placement and utilization are influenced by inter- and intra-specific factors. Bobcats are most active during crepuscular periods of the day, matching the activity of their prey [8]. Bobcats travel the longest distances at night, typically when it is coolest, and limit movement during the day [9, 10], particularly in hot climates. Male bobcats typically have larger home ranges than females to maximize breeding opportunities. Female bobcats show minimal home-range overlap with each other, and temporal partitioning when there is spatial overlap [3, 7, 9], to maximize resource monopolization and minimize interactions.

Bobcats most commonly consume lagomorphs and rodents across their range [11]. Studies have shown prey specialization by bobcats on either lagomorphs or rodents depending on interactions with sympatric competitors. In addition to their preferred prey, bobcats consume birds, mesocarnivores, and ungulates with regional variation in the relative abundance of these prey items in their diet [11, 12]. Though bobcats show within-population specialization on particular prey items [12], they are capable of exploiting a wide breadth of potential prey as dictated by regional abundance, habitat variation, and interspecific competition with other carnivores [11, 13, 14].

Studies of bobcats and other carnivore species face challenges associated with their elusive nature, relatively large home ranges, and low densities [15]. These challenges can be compounded in hot and harsh environments where additional precautions need to be taken to ensure animal welfare when conducting activities such as live trapping, which can then result in low sample sizes [3, 9, 16]. A second issue common in carnivore studies is that traditional diet analysis through morphological identification of undigested parts in carnivore scat can be biased towards larger more identifiable prey items and those for which indigestible parts are consumed [17–19]. These potential biases in how data are collected can limit or alter inference from studies on basic ecology of carnivores.

We studied bobcats at Kofa National Wildlife Refuge in the Sonoran Desert near Yuma, Arizona, USA. Our goal was to assess bobcat ecology in the desert southwest of the United States, including habitat use, home-range size, and diet composition. To evaluate habitat use and home-range size, we captured and fitted bobcats with GPS-collars, and deployed a camera-trap grid. To evaluate diet, we used next-generation sequencing-based metabarcoding to identify vertebrate prey items from bobcat scats [20, 21]. This study utilizes a complementary and robust framework to best inform our understanding of carnivore ecology.

## Results

### Trapping

Three adult females and one juvenile male bobcat were captured and fitted with Global Positioning System (GPS) collars in February and March of 2017. All four animals yielded roughly 11 months of GPS-locations (326 ± 31 days, n = 4).

### Camera trapping

Camera-traps were deployed for a total of 2506 trap days across 69 trap locations and yielded 230 photographs, comprising 96 discrete detection events, with each detection event typically containing multiple photographs. Individual identification was generally not possible from most camera-trap data given a lack of distinct variation in pelage and a high proportion of detections occurring at night, which resulted in images where sufficient sections of the body were rarely visible to determine spot patterns. Even so, we identified at least five different adult bobcats in our study area based on the camera-trap photographs, one of which was the collared male. We could not determine the sex of the remaining individuals from the angles of the photos.

### Home-range estimations and resource selection

**Fig. 1:**
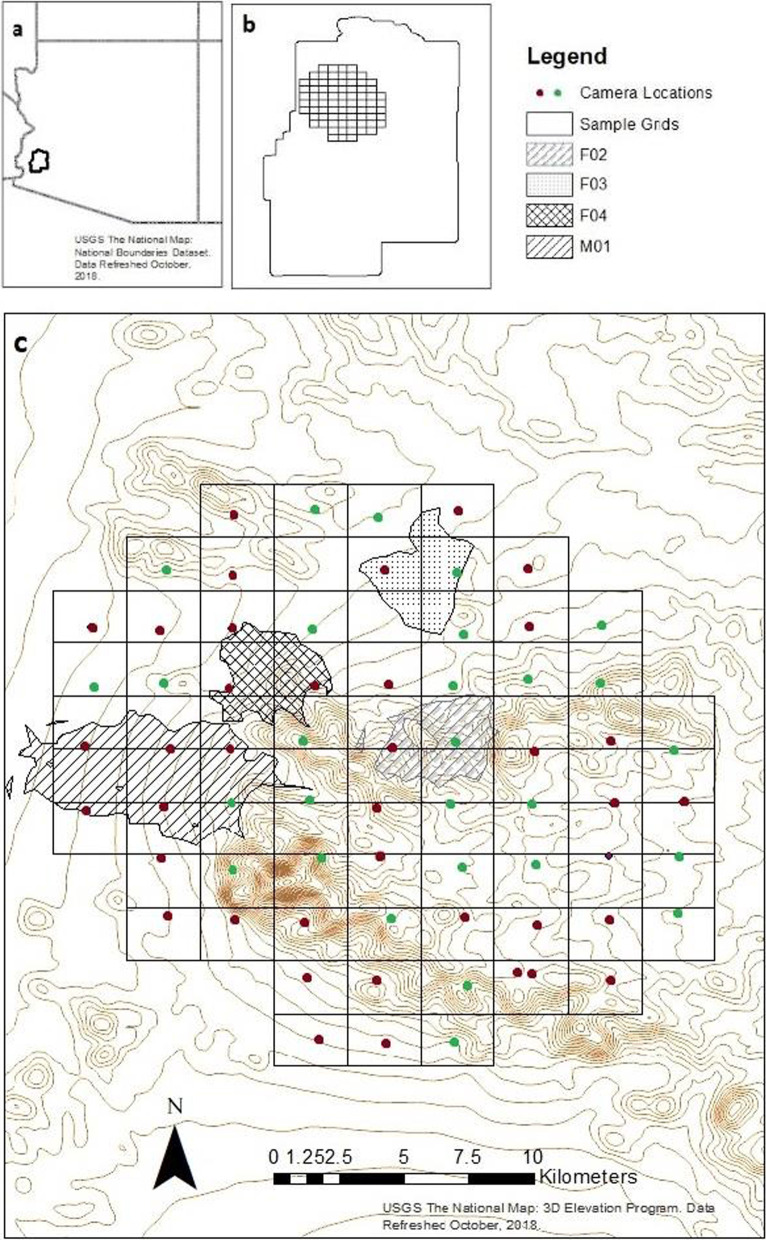
**a** The boundary of Kofa National Wildlife Refuge, Arizona, USA, **b** the 2-km sampling grid within the boundary of Kofa, and **c** the realized placement of cameras within the 69 grid cells and the home ranges of four GPS-collared bobcats. Green camera locations had at least one bobcat detection. The map data was obtained from a freely available for use source

Home ranges for the three female bobcats averaged 16.0 km^2^ (± SE 1.0 km^2^), while the single juvenile male had an annual home range of 57.8 km^2^ (Table 1, Fig. 1). When considering the sexes separately (to account for differences in effect size), home ranges were 8.3 km^2^ smaller on average during the hottest 4 months (June–September, mean temp 33 °C) of the year (p = 0.058, paired t-test) relative to the coolest 4 months of the year (Table 1, November–February, mean temp 13.5 °C) [22]. The smaller home ranges observed during the hot season were completely contained within both the home range during the cool season and the total annual home range.

**Table 1:** The 95% kernel utilization density home range sizes for each GPS-collared bobcat at Kofa National Wildlife Refuge, for the whole year (All), for the hottest 4 months of the year (Hot season, June–September mean temperature 33 °C), and for the coolest 4 months of the year (Cool season, November to February, mean temperature 13.5 °C)

ID	Home ranges (km^2^)		
	All	Hot season	Cool season
KBF02	15.2	10.6	19.4
KBF03	17.3	13.5	25.0
KBF04	14.4	11.7	16.1
KBM01	55.8	42.0	121.6
Average	25.7	19.5	45.5
Average females	15.6	11.9	20.2
SE of female HRs	0.9	0.9	2.6

The single male was a juvenile, whereas the females were adults

**Fig. 2:**
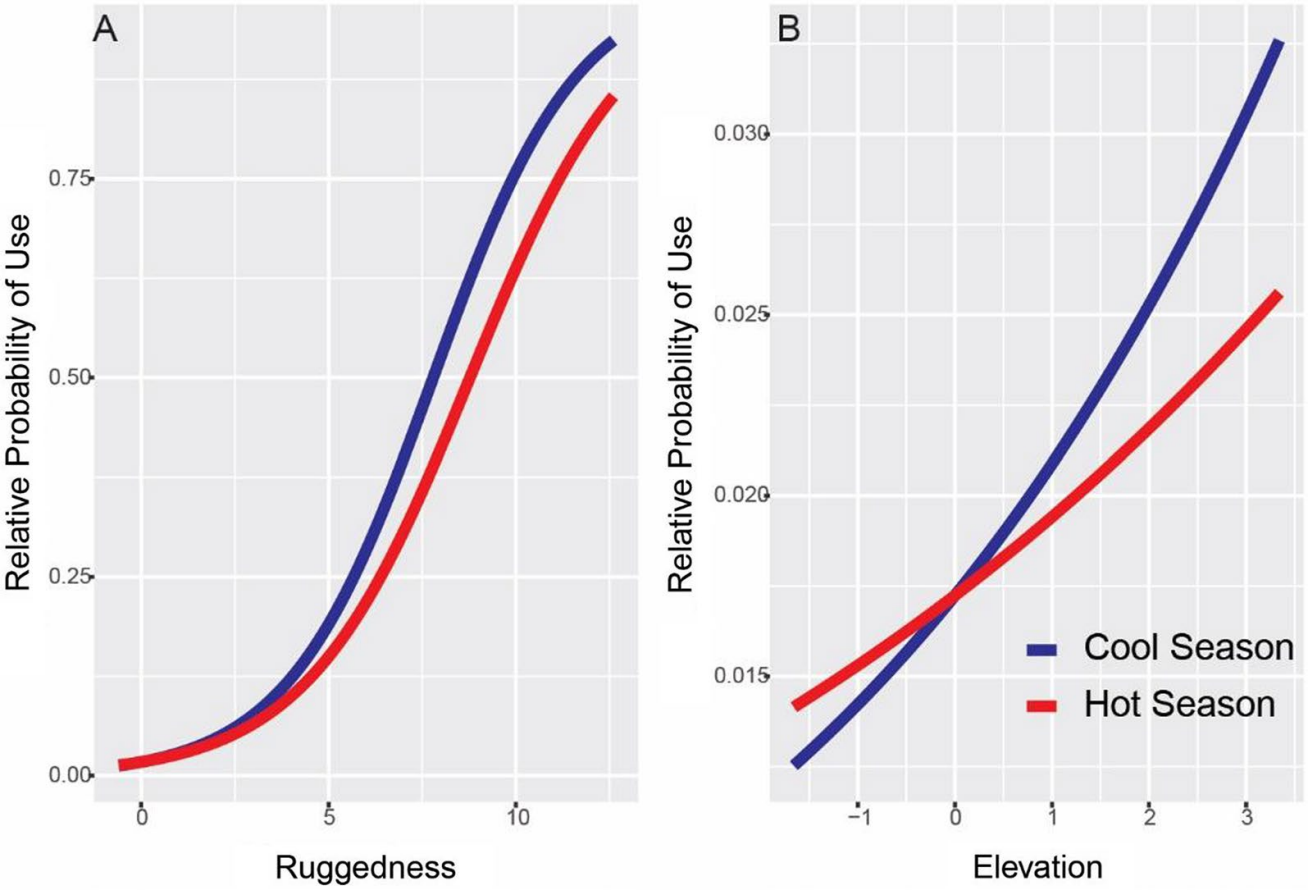
The relative probability of use by bobcats on Kofa National Wildlife Refuge as ruggedness increase away from average for hot and cool seasons, while elevation is held constant at the average for used and unused points (**A**). The relative probability of use as elevation changes for both hot and cool season while ruggedness is held constant at the average for used and unused points (**B**). The *x*-axis for both are scaled as multiples of the standard deviation away from the average, which is at 0. The extent of the *x*-axis is bounded by the maximum and minimum of each variable of observed values

All variables included in the resource selection function models had low correlation with variance inflation factors of < 2. The top resource selection function model included ruggedness, elevation, and season as well as interactions between ruggedness and elevation, ruggedness and season, elevation and season, and ruggedness, elevation, and season (Table 2). Bobcats were 1.68 times more likely to utilize rugged terrain and 1.22 times more likely to use higher elevations (Table 3, Fig. 2). The interactions between season and both ruggedness and elevation showed a slight decrease in usage during the hot season but with a large confidence interval widely overlapping zero (Table 3). Bobcats were also 0.15 times more likely to use rugged terrain as it increased in elevation. During the hot season their usage of rugged terrain as it increased in elevation was 0.53 times more likely (Table 3).

**Table 2:** AICc table for model selection of mixed effect models considered for 3rd order habitat selection by bobcats at Kofa National Wildlife Refuge, Arizona, from GPS-collar data obtained between February 2017 and January 2018

Model parameters	K	AICc	∆ AICc	AICc weight	Cumulative weight	Log likelihood
Rugged + Elev + Rugged*Elev + Rugged*Season + Elev*Season + Rugged*Elev*Season	14	32,134	0	1	1	− 16,072
Rugged + Elev + Rugged*Elev + Rugged*Elev*Season	10	32,221	88	0	1	− 16,116
Rugged + Elev + Rugged*Season + Elev*Season + Rugged*Elev*Season	12	32,567	434	0	1	− 16,289
Rugged + Elev + Rugged*Season + Elev*Season	10	32,636	503	0	1	− 16,324
Rugged + Elev + Elev*Season	8	32,709	576	0	1	− 16,361
Rugged + Elev + Rugged*Season	8	32,731	597	0	1	− 16,372
Rugged + Rugged*Season	6	32,839	705	0	1	− 16,427
Eleve + Elev*Season	6	33,020	886	0	1	− 16,518
Rugged + Elev + Rugged*Elev	8	36,878	4745	0	1	− 18,446
Rugged + Elev	6	37,124	4991	0	1	− 18,570
Rugged	4	37,159	5025	0	1	− 18,595
Elev	4	37,277	5144	0	1	− 18,655
Null	2	37,542	5409	0	1	− 18,763

**Table 3:** Coefficients for the top mixed effect model for 3rd order habitat selection by bobcats at Kofa National Wildlife Refuge and the variance of the random effect of individuals on each. Parameter values were standardized to deviation from the values mean as a multiple of the standard deviation to aid in model convergence. The average, maximum, minimum, and standard deviation of both the elevation and ruggedness for the collected data before this transformation is reported below the model estimates

Parameter	Estimate	S.E	Odds ratio	Variance of random effects
(Intercept)	− 4.04	0.30	0.02	0.36
Rugged	0.52	0.38	1.68	0.56
Elev	0.20	0.05	1.22	0
Rugged:elev	− 1.90	0.61	0.15	1.5
Rugged:hot	− 0.06	0.33	0.94	0.40
Elev:hot	− 0.07	0.50	0.93	0.98
Rugged:elev:hot	1.26	0.66	3.51	1.74

Raw data values	Average	Maximum	Minimum	Standard deviation
Elevation	609.5	1110.6	421.1	128.6
Ruggedness	3.5	78.4	0.03	5.4

### Occupancy modeling

The totality of the camera-trap grid averaged 129.5 m higher in elevation (± SE 0.49, p < 0.001, t-test) and 1.6 times more rugged (means 1.9 and 1.2, difference of 7.2 ± SE 0.007, p < 0.001, t-test) than the home ranges of the collared bobcats. Despite this more diverse coverage of terrain variables, no models reached statistical significance or an r^2^ value > 0.09 (Additional file 1: Table S1).

### Diet analysis

A total of 51 presumed bobcat scats were collected, of which 39 yielded sufficient DNA for species identification. Of the 38 samples successfully sequenced, 31 were identified as bobcat and 7 were identified as coyote (*Canis latrans*). All 31 samples identified as bobcat amplified at the 12 s and 16 s markers. The MiSeq run resulted in a total of 5,884,850 raw sequence reads. After trimming, filtering, denoising, and chimera removal with DADA2, a total of 989,861 reads remained; 594,316 belonging to 12S and 395,545 belonging to 16S. Clustering at 97% similarity resulted in a total of 32 OTUs for 12S and 20 sOTUs for 16S. Naïve lowest common ancestor analysis [23] following BLAST identified 29 of the 12S sOTUs, and 17 of the 16S sOTUs (Additional file 1: Table S2) resulting in 7 oOTUs for 12S and 11 oOTUs for 16S (Additional file 1: Table S2) after felid OTUs presumably for bobcat were removed. Samples were identified to genus or lower for 28% of 12s oOTUs and 50% of 16s oOTUs (Additional file 1: Table S2). Once oOTUs that were identified by both markers were unified, 12 dietary items were detected (Table 4). Within the 31 bobcat scats, members of the order *Rodentia* were in 28, *Passeriformes* in 21, lagomorphs in 8, and non-bobcat *Carnivora* in 6. All non-bobcat Carnivora dietary items were of the family *Canidae*; we were unable to identify one beyond family and 5 of the 6 were *Urocyon* (Table 4).

**Table 4:** Organism operational taxonomic units (oOTU) identified to their lowest taxonomic level, common name of oOTUs (*denotes common name of probable species) and the number of bobcat scats (out of a total of 31 total bobcat scats collected at Kofa National Wildlife Refuge) in which each oOTU was identified. Two oOTU’s were only identified to genus but had only one subordinate species known to be present within the study area. Each lowest oOTU is identified as being detected by the 12S or 16S marker (some were identified by both)

Total diet oOTUs	Common name	Taxonomic level	# scats	oOTUs 12S	oOTUS 16S
*Ammospermophilus harrisii*	Harris antelope squirrel	Species	1		Yes
*Chaetodipus baileyi*	Bailey's pocket mouse	Species	8	Yes	Yes
*Chaetodipus californicus*	California pocket mouse	Species	1		Yes
*Neotoma lepida*	Desert woodrat	Species	2		Yes
*Mus* (subgenus)	Mice	Subgenus	1		Yes
*Lepus*	Black-tailed jackrabbit*	Genus	8		Yes
*Neotoma*	Pack rats	Genus	1		Yes
*Urocyon*	Gray fox*	Genus	5	Yes	
*Canidae*	Canids	Family	1	Yes	
*Cricetidae*	New World rats and mice, hamsters, voles, lemmings	Family	8		
*Muroidea*	Mice, rats, voles, hamsters, gerbils	Super family	17		Yes
*Passeriformes*	Perching birds	Order	21	Yes	Yes

## Discussion

Our results illuminate bobcat ecology in a harsh desert climate. Bobcats on Kofa appear to utilize rugged terrain and higher elevations, and consume mostly small prey items. Bobcats also show seasonal variation in space and habitat use; they avoid rugged terrain at higher elevations during the cool season and decrease their home-range size during the hot season. Concurrent sampling strategies and the use of modern technology in diet analysis allowed for these conclusions despite harsh sampling conditions impeding our design and sampling efforts.

Our study is one of only a few to examine home ranges and space use of desert bobcats [3, 9, 10], leaving much room for further exploration of bobcats in hot, arid climates. The female home ranges in this study were substantially smaller (16.0 km^2^ ± 1.0 SE) than an average of 29 studies of bobcat space use in their more northern range or for bobcats in the Chihuahuan Desert (23.9 km^2^ ± 4.2 SE, 27.1 km^2^ ± 6.4 SE, respectively) [9]. While we are unable to generalize from the estimate of home-range size from one male, it is interesting to note the male was young and his home range size may have been larger than other males if he had not yet established a territory. Although our sample sizes were small and limit our power to generalize our gross home-range size findings, the within-individual comparison of seasonal home-range size supports our expectations regarding movement reductions in the hot season. There was a substantial and significant reduction in home-range size between the cool (20.2 km^2^ ± 2.6 SE, 13.5 °C) and hot seasons (11.9 km^2^ ± 0.8 SE, 33 °C). This differs from the lack of a significant difference in seasonal home-range size between bobcats in the Chihuahuan Desert and studies of more northern populations [9]; however, the portion of the Chihuahuan Desert sampled averaged 9 °C cooler in the winter and 5 °C cooler in the summer compared to the Sonoran Desert. The smaller home ranges that we found in the hot season suggest bobcats in extremely hot climates reduce their movements seasonally in response to increases in temperature, which has also been observed in other carnivore species [24]. We hypothesize that this response is due to a reduction of available surface water and an increase in water needs for long-distance movement and thermoregulation. Future studies on desert-dwelling bobcats that can compare between sexes and age classes would elucidate the relationship between water, thermoregulation, and space use. Unfortunately, we were unable to accomplish this analytical step due to our limited sample size.

In addition to small sample size, another limitation to our analysis is that we only recorded locations during crepuscular and nocturnal time periods that may have biased our assessment of space use. However, previous research has found that the vast majority of desert bobcat movement takes place during these time periods [9, 10]. Therefore, it is highly unlikely that the exclusion of daytime locations prevented the detection of movements that would alter home-range size estimates.

Although our camera grid provided reasonable detection rate for an elusive carnivore, the models failed to adequately describe occupancy trends; however, third-order habitat selection from GPS-collar data provided some insight into habitat use of desert bobcats. Bobcats favored higher elevations or rugged terrain at lower elevations. While results of our top model should be considered cautiously because of the low sample size of GPS-collared bobcats, selection of rugged terrain and higher elevations by bobcats in this environment is realistic. Bobcats may use rugged terrain to avoid humans, who are more likely to use lower elevations and less rugged terrain (e.g., [25]), avoid coyotes [26], or to follow prey distribution [27]). Bobcat preference for higher elevations is likely to seek out cooler temperatures, while avoiding high elevation rugged terrain that would be energetically costly to traverse and relatively absent of vegetative cover. During the hot season, the cooler temperatures found at higher elevations likely outweigh the costs of using higher elevation rugged terrain. Within the home ranges of the four GPS-collared bobcats, low-elevation, rugged terrain (i.e., mostly dry washes in the flatlands between mountain ranges) were likely utilized as travel corridors and rest locations. These washes provide the only meaningful cover on the lower elevation landscape for both bobcats and their prey. This finding aligns with previous research that has looked at desert bobcat space use [3].

It is interesting that ruggedness appeared in our top models even though we did not initially capture the bobcats in rugged or high terrain. For timely animal processing and release, we limited live trapping to lower, less rugged terrain with almost no opportunities to set traps in higher, rugged terrain that was more commonly accounted for within the camera-trap survey. Had detection been higher within the camera-trap grid, it would have been possible to examine habitat use of bobcats who were exposed to more rugged terrain in high elevation. This limitation supports the need to account for the elusive nature of carnivores and the need for longer sampling periods and complimentary sampling methodologies to obtain information on space use by desert carnivores.

Our study contributes to the growing body of literature using metabarcoding for diet analysis and is the first to utilize this technique for bobcats. Nearly all scats collected included *Rodentia* DNA (83%) and more than two-thirds of scats collected included *Passeriformes* DNA (68%). Frequency of occurrence of rodents was similar to that observed in other bobcat populations [1, 11, 12, 28, 29]. Slightly larger prey items such as *Urocyon* and *Lepus* species were detected in ≤ 25% of scats. However, these larger prey items were not detected in scat in absentia of DNA from other prey items, suggesting that capture of these larger prey items was opportunistic while pursuing a diverse prey base. It is also possible that foxes were detected by metabarcoding because they urinated on top of bobcat scats, and were not in fact a prey item. This behavior has been demonstrated in coyotes contaminating scats of cougars (*Puma concolor*) [30]. We did not detect any larger-bodied species such as desert bighorn sheep or mule deer, despite scats being collected during fawning and lambing seasons [31, 32]. Consistent with our results, deer likely represents only a minor food item in desert regions [1, 33], even though studies in more northern climates show deer to represent a more substantial food item in bobcat diet [12, 29]. Our findings on Kofa also support previous studies’ findings that bobcats were likely not a source of mortality for desert bighorn sheep [32]. This may be important to bighorn sheep management.

The main difference between our diet findings and previous studies is the high relative frequency of avian species. In previous studies, bird species were limited mostly to < 10% of relative frequency and, at most, 31% of relative frequency [1, 11, 12, 28, 29]. These differences could be due to differences in foraging strategies among bobcat populations, or the differences in methodology that were used to detect prey [33]. Previous research has suggested that smaller animals, such as birds, have the largest potential for error in traditional morphology-based, post-ingestion diet analysis [17, 33] because they are digested beyond the ability for visual identification. Therefore, we believe that the higher proportion of avian species in our diet analysis is likely due to improved detection rather than higher consumption rates compared to other environments. Notably, we were able to assign most sequence reads at the species or genus level with both markers for mammalian prey. However, we were only able to assign avian reads at the order level. Although the pan-vertebrate 12S marker used in this study seems to amplify avian prey well, this marker appears to have poor taxonomic power for assigning reads at higher taxonomic levels for birds. The region of 12S mitochondrial DNA targeted by this marker appears to have little variability within avian species, and particularly for species within the order Passeriformes. Thus, family, genus, and species level assignments were not possible using this marker. Given this limitation, future studies should consider adding an additional, avian-specific marker if more precise taxonomic identity of avian prey is desired. Increased use of metabarcoding for diet analysis is needed to further explore differences between ecosystems versus differences between sampling methodologies.

## Conclusions

In this study, we found that desert bobcats have smaller home ranges that vary seasonally and consume little to no large prey items relative to bobcats studied in more northern regions. The use of new technology allowed for identifying previously undercounted diet items (i.e., avian species in bobcat diet) that can be important for understanding the totality of bobcat predation and specialization. We encourage other researchers to combine multiple, modern techniques when conducting field studies, especially in regard to studies of rare and elusive species in harsh environments. Extending the sampling period to would likely also be beneficial. The differences in space use and diet we observed were only possible through the use of multiple field and laboratory techniques and likely reflect adaptations to living in a harsh environment.

## Methods

### Study area

Kofa National Wildlife Refuge (hereafter referred to as Kofa) is located in southeastern Arizona, USA (Fig. 1), with an annual average rainfall of 10 cm, an annual mean temperature of 22 °C, and an average monthly maximum of 37 °C [22]. Kofa is sparsely vegetated with desert-adapted species. The refuge includes two mountain ranges, the Kofa Mountains and the Livingston Hills, with relatively flat lowlands between them. Terrain features were of particular interest in this study as the availability of escape terrain has been identified as an important influence on the space use of potential prey species in the area [28]. Multiple potential prey species for bobcats exist including 19 species of *Rodentia* and two species of *Lagomorpha*, desert bighorn sheep (*Ovis canadensis mexicana*), Sonoran pronghorn (*Antilocapra americana sonoriensis*), and mule deer (*Odocoileus hemionus*), and over 150 species of birds.

### Trapping

We set traps for bobcats on Kofa in the lowlands between the Kofa Mountains and the Livingston Hills during the cool season of 2016–2017. All bobcats were captured in accordance with Arizona Game and Fish Department (AZGFD) permits and protocols, by AZGFD personnel, utilizing Victor 3N soft-catch foot-hold traps equipped with remotely enabled trap alerts that notified personnel when a trap was tripped. Additionally, all traps were visually checked every 12–24 h. Traps were placed near dirt roads to ensure quick access when a trap-closed alert was sent and spaced to capture bobcats likely to have neighboring home ranges. Captured animals were chemically immobilized utilizing a combination of ketamine (10 mg/kg) and xylazine (1.5 mg/kg) [16]. Once immobilized, each bobcat was fitted with a GPS-enabled collar equipped with remote upload (Vectronic Aerospace, Berlin Germany). Collars were scheduled to take daily locations every 4 h between 8 pm and 8 am to capture nocturnal movement while sustaining battery power for at least 1 year.

### Camera trapping

We also deployed camera traps on a 2-km grid across ~ 300 km^2^ of Kofa. The grid included all elevations and terrain types. Two game cameras (Browning Strikeforce HD Pro X) were placed opposing each other across likely wildlife trails within each grid cell while maintaining relational spacing with other cameras (Fig. 1). Camera were set to a burst of three photographs with a 30-s delay between bursts and have Infrared LED for nighttime photographs. Cameras were active at each site for 5 weeks, which constituted a camera-trap session. Cameras were deployed in three successive 5-week intervals, to allow for logistical and resource constraints, resulting in all 69 grid points were sampled during the cool season (December 2016 and May 2017).

### Home-range estimations and resource selection

Location data obtained from the GPS collars in 2017 were used in a mixed effect model of third order resource selection (individual selection within their home range) [34]. The first 72 h of location data from each individual was removed to avoid any anomalous post-capture movements. All fixes with a dissolution of precision ≥ 10 were removed [35]. Terrain values (elevation and ruggedness: the absolute mean difference between the elevation of a cell and its surrounding cells [36]) were extracted and calculated from USGS elevation data aggregated in the terrain data set available from ESRI at a 30-m resolution [37]. Variables were evaluated for correlation with a variance inflation factor threshold of ten to prevent collinearity in model slopes [38]. Location-specific values were extracted from these layers for each collar location, all values were standardized (mean = 0, SD = 1) using all used and unused values to aid in model convergence. Home ranges were generated for each individual utilizing a 95% kernel utilization distribution [39] an h_ref_ bandwidth estimator was used to reduce fragmentation of the home range [39]. Home ranges were estimated with the package adehabitatHR in the R environment [40, 41]. Terrain variables were extracted for each home range at 30-m intervals to quantify third order available habitat, this resulted in greater than 14 available points to every used point for the four individuals. Land cover within the home ranges where third-order habitat selection was being assessed was homogenous with over 99.7% being classified as shrub/scrub at a 30-m resolution by the National Land Cover Database [42]. Multiple binomial mixed effect (used vs available) models were generated to explore the effect of terrain features on third-order habitat selection using the lme4 package in R [43]. All models included random intercepts and slopes to account for individual variation in habitat use and home-range size and composition [44, 45]. Perennial water sources were removed from consideration because mapped perennial water sources only existed within the home range of a single collared bobcat, therefore lacking replication. Additionally, water resources of the other three bobcats (and possibly additional sources for the one that overlapped a mapped water source) were likely unmapped or ephemeral sources that could not be accounted for in this analysis. The models that produced significant fixed effects (α = 0.05) were then evaluated using Akaike’s Information Criterion, corrected for small sample size (AICc) [46] to select a top model for inference.

### Occupancy modeling

Occupancy modeling was carried out in program RMark [41, 47] utilizing data from the camera trap grid to establish occupancy across all of Kofa. Elevation and ruggedness data were generated from the same sources using the same methods as used for the GPS-collar data. Ruggedness and elevation were then averaged over 510 m to characterize the terrain traveled through to reach the camera as well as the immediate terrain. Values were standardized to deviation from the values mean as a multiple of the standard deviation to aid in model convergence. Single season occupancy models were then generated utilizing terrain factors that could influence detection and occupancy [48]. Models were evaluated utilizing both 30- and 510-m resolutions of ruggedness and elevation in varying combinations. Models were evaluated for significant fixed effects at a α of 0.05.

### Diet analysis

Within each cell of the camera grid, a likely travel corridor (e.g., road, trail, dry creek bed) was identified and utilized as a representative 1-km transect for the cell. Each transect was first cleared of all carnivore scats prior to the experimental period to ensure only fresh scat were collected, then walked once. All collected scats were frozen and stored in a − 20 °C freezer to preserve DNA quality. Extraction of DNA for species identification was carried out using Qiagen stool kits following a protocol modified from the manufacturer’s standard protocol (Qiagen Inc., Valencia CA, Appendix 1). Each scat was identified to carnivore species by sequencing a 126 bp mini-barcode fragment or the mitochondrial gene ATP6 [49]. Once species was confirmed, a second extraction was run on each bobcat scat using a homogenized cross-section of the scat to better capture prey DNA (Appendix 1).

In order to genetically identify prey items from scat, we performed DNA metabarcoding [50]. Libraries for next-generation sequencing were prepared using a two-step PCR protocol for two loci: a mammal-specific primer set (16Smam1, 16Smam2) [51] targeting 130–138 bp (including primers) of the mitochondrial 16S rRNA locus, and a pan-vertebrate primer set (12SV5F, 12SV5F) [52] targeting 140–143 bp (including primers) of the mitochondrial 12S locus. Initial PCR Reactions were conducted in duplicate for each locus, with one replicate containing bobcat-blocking primers designed for this study and one replicate containing no bobcat-blocking primers. All replicates contained human blocking primers. Replicates from the initial PCR for each sample were pooled prior to the second PCR used to add dual indexes for sample identification. Information on primers and PCR conditions can be found in Appendix 1. Samples were then sequenced on an Illumina MiSeq with a 300 cycle V2 micro kit.

Following demultiplexing, primer sequences were removed and 12S and 16S sequences were separated using CUTADAPT v. 1.18 [53]. Next, data were filtered, denoised, paired-ends were merged, and chimeras were removed with DADA2 [54] within the QIIME2 v. 2018.8 environment [55], and reads were truncated at 108 bp (12S) and 94 bp (16S). Sequences were then clustered into sequence operational taxonomic units (sOTU) at 97% similarity using q2-vsearch in QIIME2. sOTUs were run through NCBI BLAST to identify probable assignments that were then analyzed using a naïve lowest common ancestor algorithm in MEGAN [23] to assign an organism operational taxonomic unit (oOTU).

## Supplementary Information

**Additional file 1: Table S1.** Occupancy model results from camera traps. **Table S2.** BLAST # constructed from biom file.

**Additional file 2.** DNA extraction modifications from Qiagen Qiaamp DNA Stool mini Kit instructions.Modifications listed by corresponding step from Qiagen instructions, un-listed steps remain unchanged.

**Additional file 3: Additional Data.** All raw data and details from camera trap models are available as Additional file 3.

## Data Availability

All data generated or analysed during this study are included in this published article in its Additional files 1, 2, 3.

## Abbreviations

GPS: Global Positioning System
Kofa: Kofa National Wildlife Refuge
oOTU: Organism operational taxonomic units
sOTU: Sequence operational taxonomic units
